# Data on the function of CDH17 in pancreatic cancer growth

**DOI:** 10.1016/j.dib.2019.104332

**Published:** 2019-08-13

**Authors:** Hao Yuan, Joseph Stenberg, Guangfu Li

**Affiliations:** a Department of Surgery, University of Missouri, Columbia, MO, 65212, USA; b Ellis Fischel Cancer Center, University of Missouri, Columbia, MO, 65212, USA; c Pancreas Center, The First Affiliated Hospital of Nanjing Medical University, Nanjing, Jiangsu, 210029, China; d Molecular Microbiology and Immunology, University of Missouri, Columbia, MO 65212, USA

**Keywords:** Pancreatic cancer, CDH17, Tumorigenesis

## Abstract

Data presented in this article are related to the research article entitled “Disruption of oncogenic liver-intestine cadherin (CDH17) drives apoptotic pancreatic cancer death”. To investigate the influence of CDH17 on human pancreatic cancer (PC), we performed gain and loss of CDH17 function with siRNA and recombinant plasmid to evaluate its impact on PC cell proliferation, colony formation, and migration. The data can be valuable for researchers interested in the study of oncogenic activity related to the CDH17 gene in PC growth and motility.

**Table:** Specifications Table

Subject area	*oncology*
More specific subject area	Pancreatic cancer, tumorigenesis
Type of data	Image, graph, and figure
How data was acquired	Microscope (Zeiss, Vert A1), mass spectrometry (Molecular Devices, iD3), GEPIA (Gene Expression Profiling Interactive Analysis: gepia.cancer-pku.cn/).
Data format	Raw Analyzed
Experimental factors	CDH17 was demonstrated as an oncogene to impact pancreatic cancer growth.
Experimental features	Knockdown or overexpression of CDH17 on cell proliferation, colony formation, and migration.
Data source location	Department of Surgery in the University of Missouri, Columbia, MO 65212, United States
Data accessibility	Data are in this article
Related research article	Liu, X. et al. Disruption of oncogenic liver-intestine cadherin (CDH17) drives apoptotic pancreatic cancer death Cancer Letters 10.1016/j.canlet.2019.04.022

**Table:** 

**Value of the data** • The data presented here was acquired to assess any influence of CDH17 on human PC. Therefore, the data might contain valuable information on the clinical significance of CDH17 in human cancer. • Beneficiaries of these data include those searching for new therapeutic targets for PC or those seeking greater understanding through mechanistic studies. • These data are the first to report the effect of CDH17 on PC. Further, the methods used may provide further insight for the exploration of other therapeutic targets for cancer.

## 1 Data

**Fig. 1:**
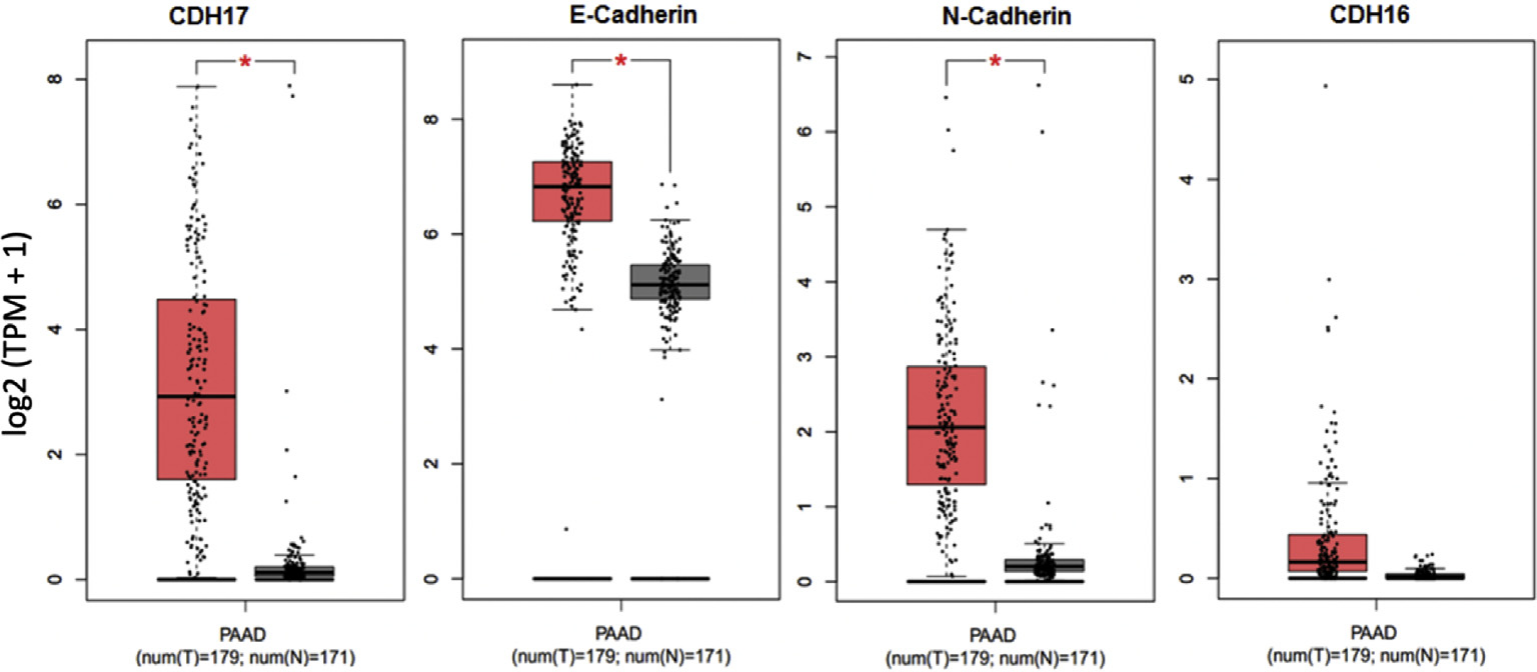
**The mRNA expression of CDH17 and other cadherin family members in human PC tumors.** The data are from open online database GEPIA. The mRNA levels of CDH17 and other cadherin family members including E-Cadherin, N-Cadherin, and CDH16 in human PC tumors from 179 patients were detected and compared to that in healthy human pancreas in 171 subjects. The boxplot analysis used log2 (TPM + 1) for log-scale.

**Fig. 2:**
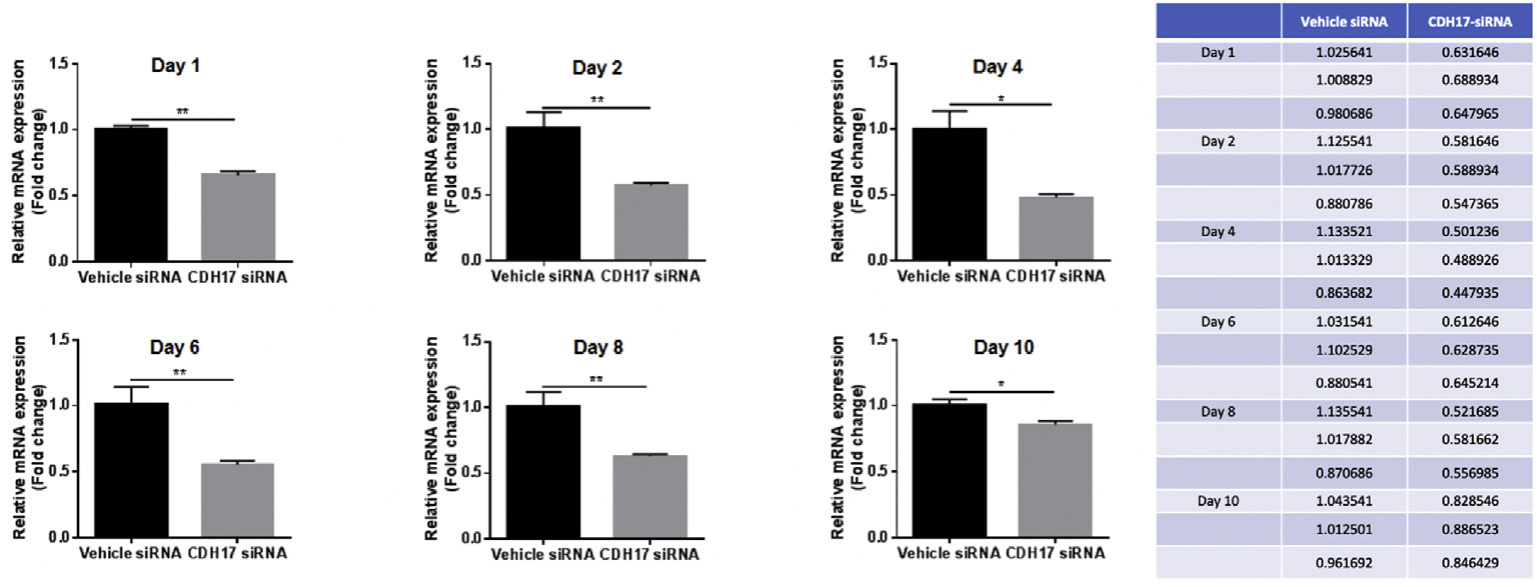
**The lasted efficacy of siRNA-mediated CDH17 knockdown.** As described in the materials and methods, Panc02-H7 cells were grown to 50% confluence in 6-well plate, then transfected with 5 pmol of siRNAs by using RNAi-MAX Lipofectamine reagent. The cells were harvested over the indicated times post-transfection to extract total RNA. qPCR detected CDH17 suppression even ten days after siRNA transfection. n = 3, error bars represent mean ± SD.

**Fig. 3:**
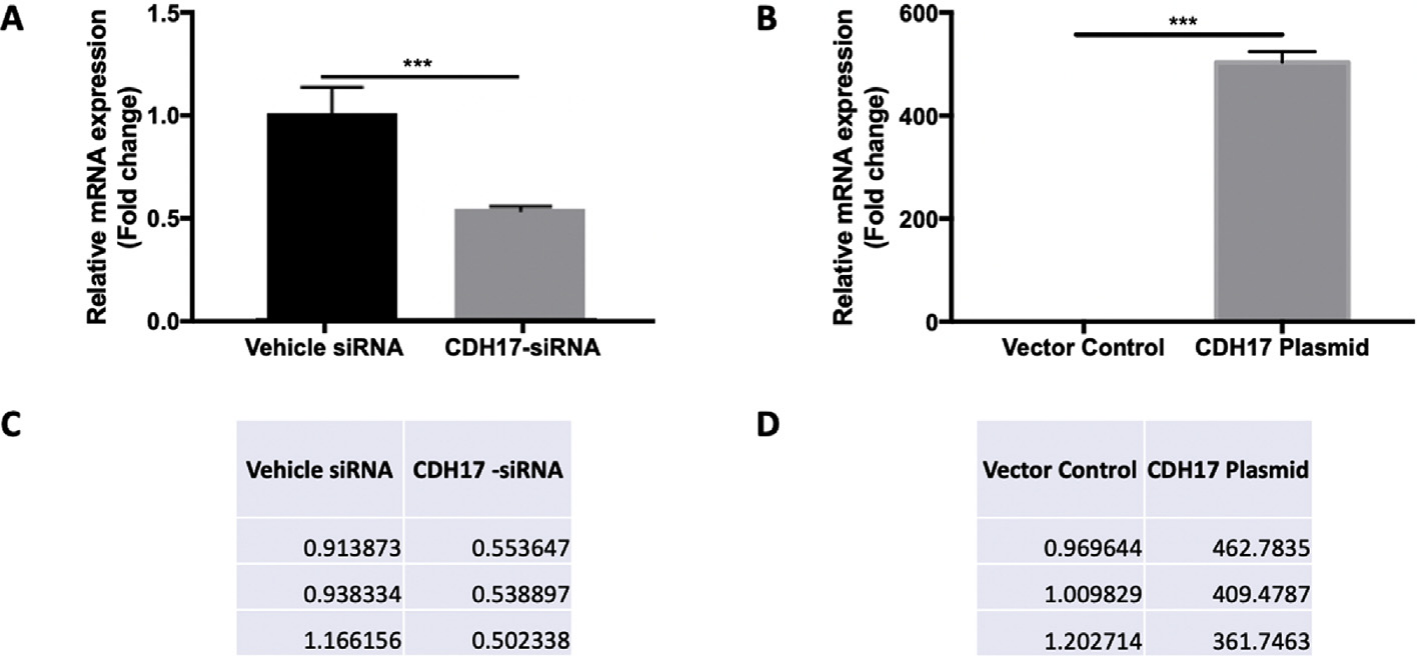
**siRNA-mediated knockdown or recombinant plasmid-mediated ectopic expression of CDH17 in PC cells.** The indicated cells grown to 50% or 90% confluence in 6- well plate were transfected with siRNA or recombinant plasmids respectively. 48 hours post-transfection, the indicated cells were harvested to extract total RNA. qPCR detected the reduced CDH17 mRNA expression in siRNA transfected human Panc-1 (A) cells as well as dramatically increased CDH17 expression in plasmid-transfected Panc-1 (B) cells. n = 3, error bars represent mean ± SD.

**Fig. 4:**
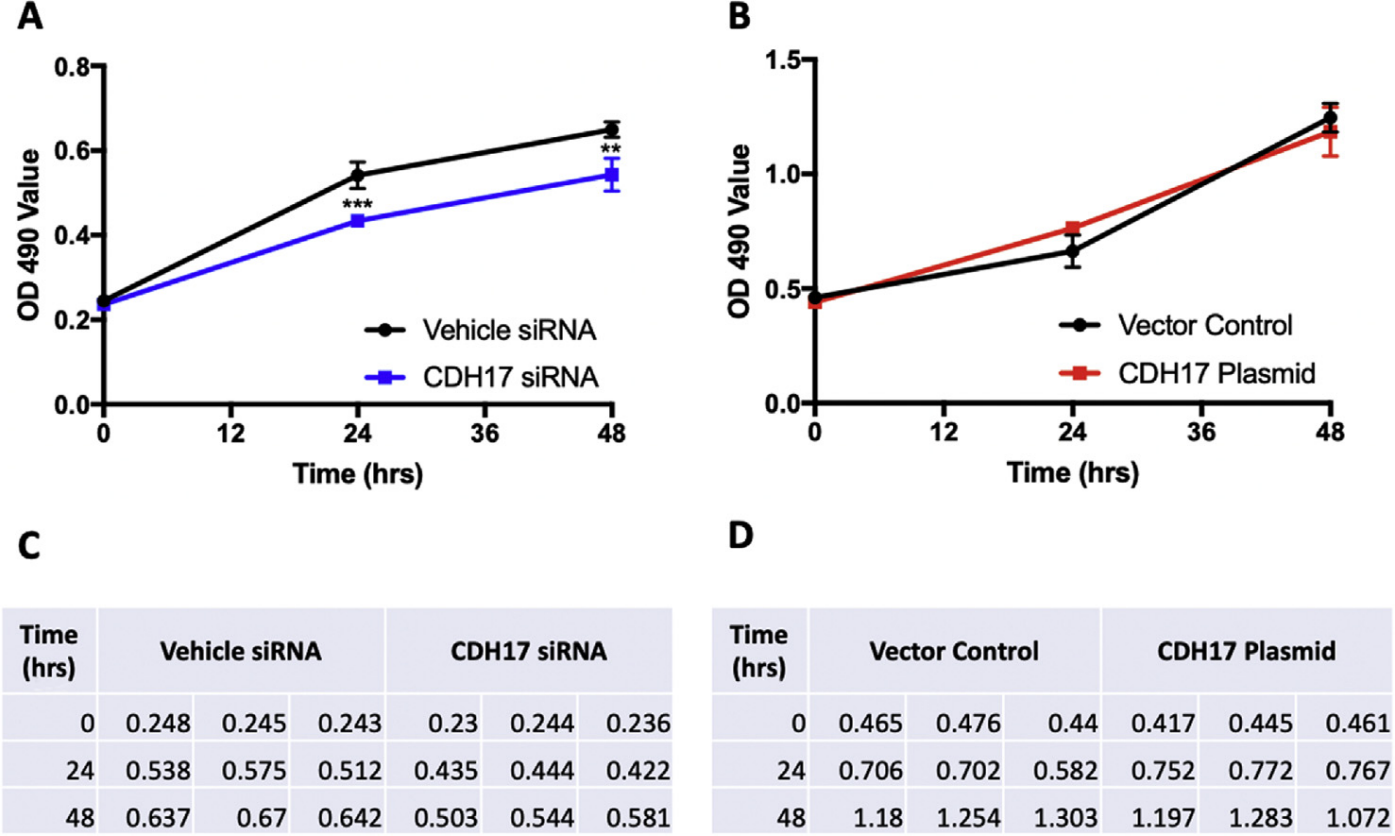
**CDH17 knockdown and overexpression impact human PC cell proliferation.** Human Panc-1 cells with siRNA-mediated CDH17 knockdown or recombinant plasmid-mediated CDH17 overexpression were seeded in 96-well plates. One or two days later, the cell proliferation was measured with Promega Proliferation Assay Kit for human Panc-1 (A and B) cells.

**Fig. 5:**
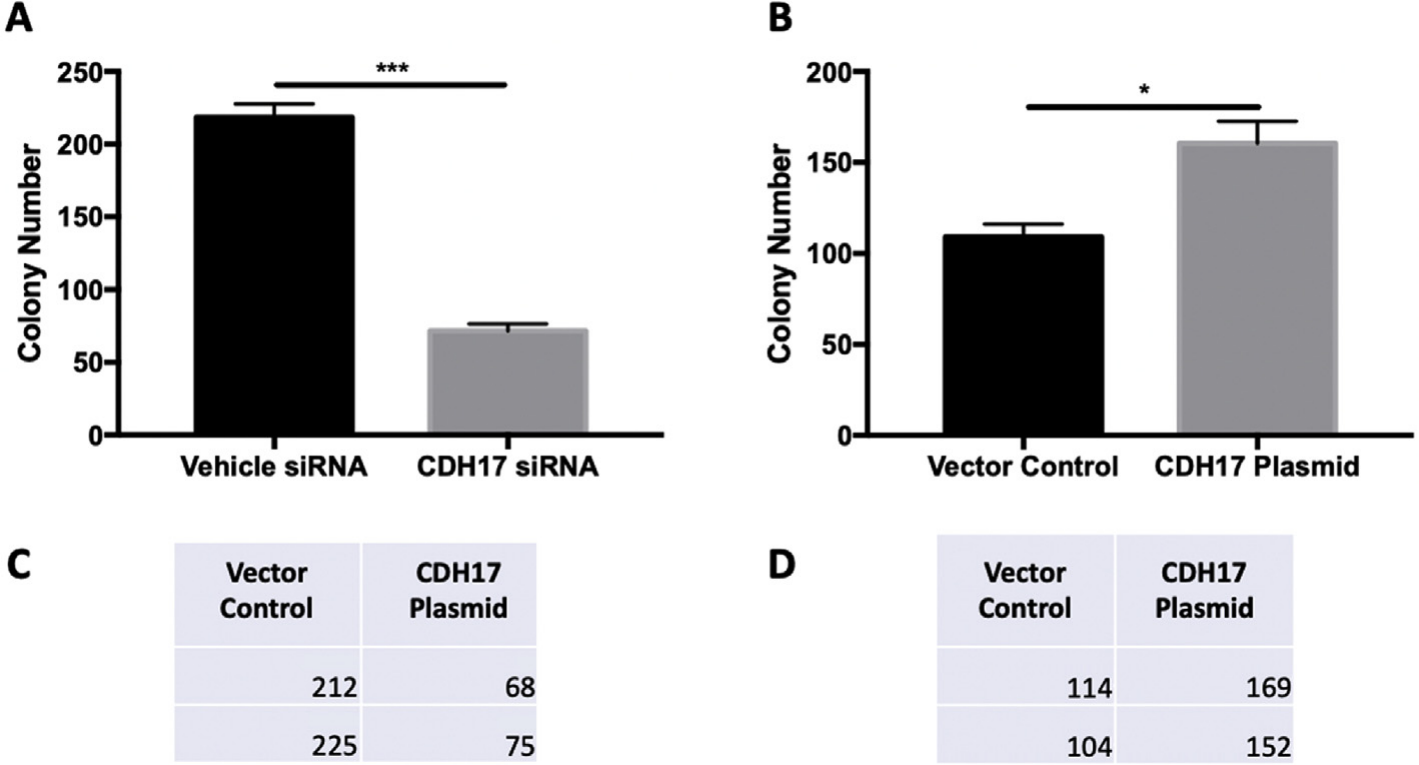
**CDH17 knockdown and overexpression impact human PC cell clonogenic formation.** Human Panc-1 cells with siRNA-mediated CDH17 knockdown or recombinant plasmid-mediated CDH17 overexpression were seeded in 6-well plates. Ten days later, cell colony formation was measured for the Panc-1 cells (A and B).

**Fig. 6:**
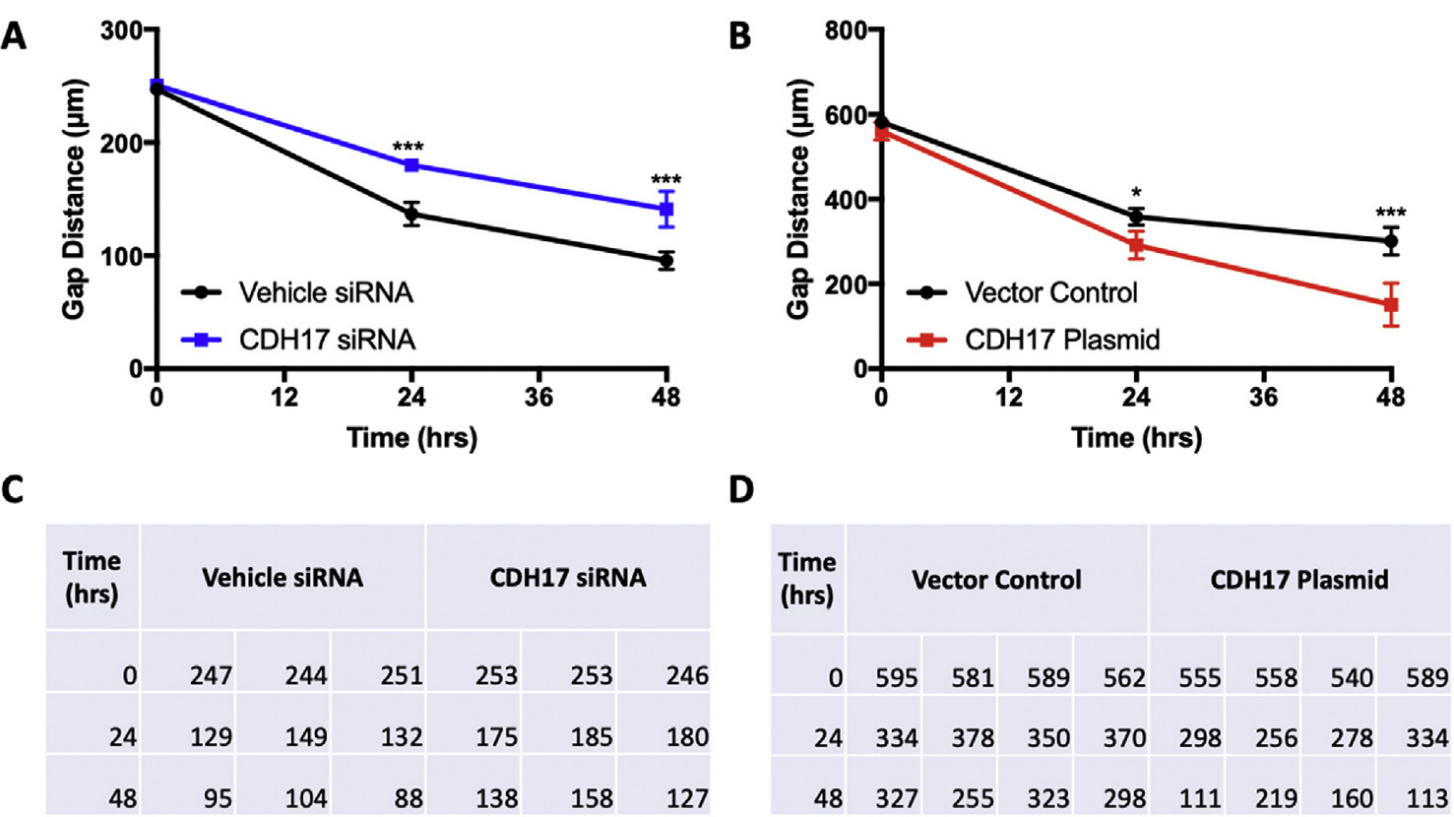
**CDH17 knockdown and overexpression impact human PC cell motility.** Human Panc-1 cells with siRNA-mediated CDH17 knockdown or recombinant plasmid-mediated CDH17 overexpression were seeded into 24-well plate at a dose of 2 × 10^5^ cells/well. The insert in each well was removed on the second day. The cell-free gap was measured at the indicated times.

**Fig. 7:**
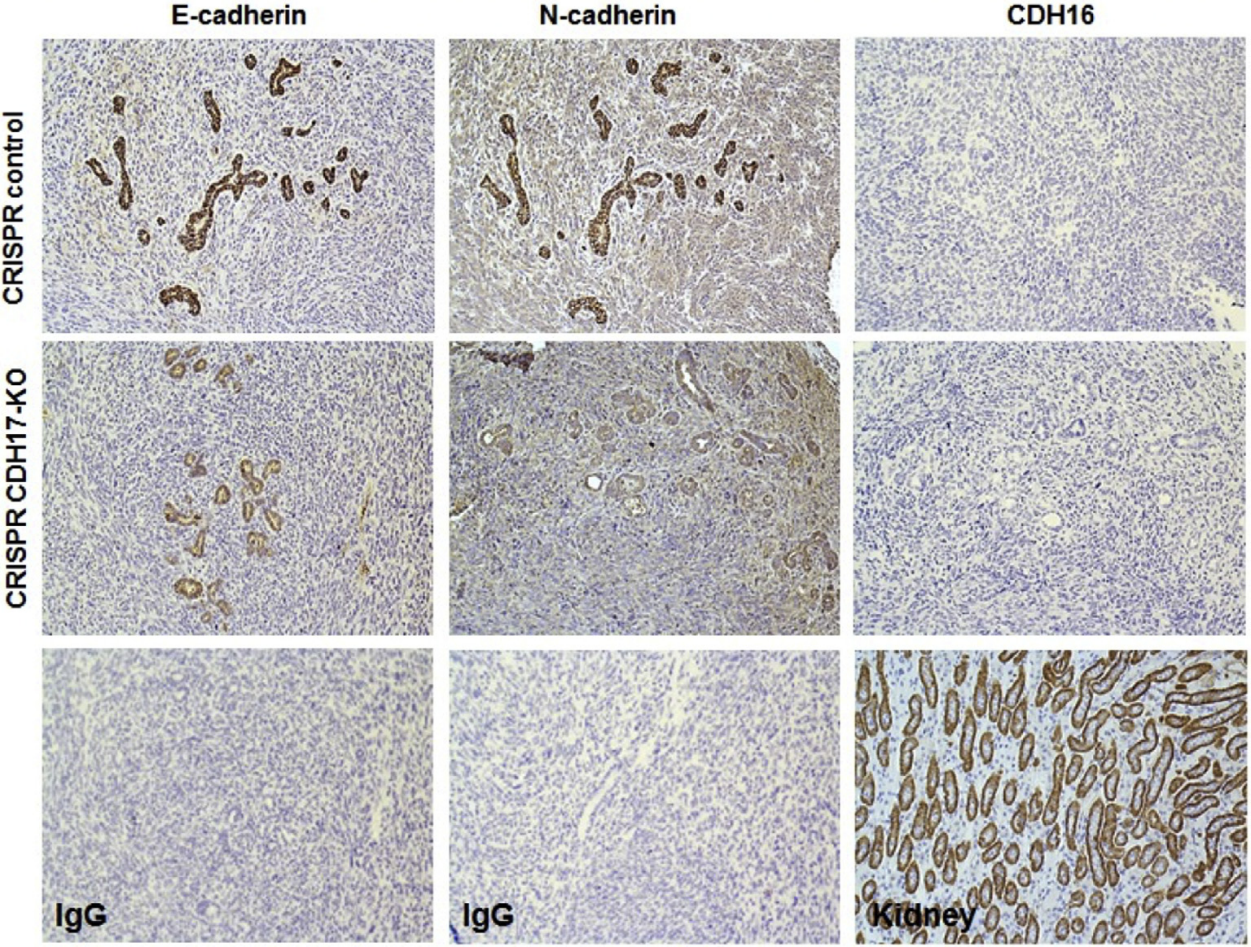
**The expression of cadherin family members in mouse PC tumors and its correlation with CDH17.** Orthotopic tumors derived from CDH17-knockout Panc02-H7 cells and their control cells were harvested and sectioned as described in materials and methods. IHC was used to detect the expression of cadherin family members including E-cadherin, N- Cadherin, and CDH16 (upper panel). The impact of CDH17 knockout on the expression of other cadherin family members was shown in the middle panel. Negative control for E-Cadherin and N-Cadherin by staining tumor section with isotype IgG as well as a positive control for CDH16 by staining kidney section with high expression of CDH16 was shown in lower panel.

The shared data are a summary of the impact of CDH17 in PC through in various assays. The mRNA expression of various cadherin family members, including CDH17, in PC and compared to healthy pancreatic tissue was taken from the online database GEPIA (Fig. 1). Using the results from GEPIA, the CDH17 gene was selected for further investigation. siRNA-mediated CDH17 knockdown was performed in mouse Panc02-H7 cells to determine the lasted efficacy of the siRNA treatment over the course of 10 days (Fig. 2). CDH17 knockdown and overexpression was then performed in human PC cell line Panc-1 and mRNA expression of CDH17 measured for treatment performance (Fig. 3). Gain and loss of function was used to investigate the impact of CDH17 on human PC cell proliferation (Fig. 4), cell colony formation (Fig. 5), and cell migration (Fig. 6) using the Panc-1 cell line. The mouse Panc02-H7 cell line underwent CDH17 CRISPR-mediated knockout for seeding into WT C57BL/6 mice. After the orthotopic tumors formed, cancerous tissue was extracted for IHC of cadherin family members (Fig. 7).

## 2 Experimental design, materials, and methods

Below is a brief description of the experimental methods used to acquire data in this paper. For a more detailed and thorough report, please refer to the related research article [1].

### 2.1 Cell culture

The mouse panc02-H7 cell line was a gift from the MD Anderson Cancer Center. The human cell line Panc-1 was purchased from the American Type Culture Collection (ATCC). The panc02-H7 cell line was maintained in Dulbecco's Modified Eagle Medium (DMEM; Cellgro) containing 100 U/mL penicillin, 100 μg/mL streptomycin, 2 mmol/L L-glutamine, 10 mmol/L HEPES, and 10% fetal bovine serum (FBS) at 37 °C in a 5% CO_2_ humidified incubator. Panc-1 human cell line was cultured in DMEM containing 10% FBS, 2.5% equine serum, 100 U/mL penicillin, and 100 μg/mL streptomycin at 37 °C in a 5% CO_2_ humidified incubator.

### 2.2 CDH17 siRNA transfection

Mouse and human cell lines were grown to 50% confluence, then underwent siRNA transfection with RNAi-MAX Lipofectamine reagent (Invitrogen). Eight hours post-transfection medium was replaced with fresh complete medium. The designed mouse or human CDH17-siRNAs, and their negative control siRNAs, were purchased from IDT.

### 2.3 CDH17 recombinant plasmid transfection

Human PC cells were grown to 70% confluence, and then received 2.5 μg human CDH17 recombinant plasmid (RC211298, OriGene) or negative control plasmid (PS100001, Origene) transfection with Lipofectamine 3000 reagent (Thermo Fisher Scientific). Eight hours post-transfection medium was replaced with fresh medium. Transfection was validated through qPCR using the VP1.5 and XL39 primers provided by OriGene.

### 2.4 CRISPR mediated CDH17 knockout

LentiCas9-EGFP plasmids were transfected into Panc02-H7 cells for establishment of Cas9-expressing cell clones. The crRNAs and tracrRNAs used were designed online (http://crispr.mit.edu) and synthesized through IDT. crRNAs and tracrRNAs suspended in nuclease-free water were heated to 95 °C and then cooled down to room temperature in order to form guide RNA duplex (gRNA). The formed gRNA, 5′-GATGATCCGGCTACTCCCAATGG-3′, was transfected into Cas9-Panc02-H7 stable cells using the RNAi-MAX Lipofectamine (Invitrogen). Single cell clones were generated with limited dilutions. The genomic DNAs were extracted from each clones with SV genomic DNA kit (Promega). PCR was used to amplify CDH17 DNA fragments with specified primers. These DNA fragments were digested using an endonuclease for evaluation of cutting efficiency mediated by Cas9 and the gRNA. Sanger sequencing was performed to identify the base-depleted site in the targeted DNA fragment. qPCR was used to measure CDH17 mRNA expression in each CDH17 knockout clone.

### 2.5 Total RNA extraction and qPCR

Trizol reagent (Invitrogen) was used to extract total RNA from the cells. mRNA was reversely transcribed into cDNA with the High Capacity cDNA Reverse Transcription Kit (Applied Biosystems). Standard SYBR Green I Real-time PCR was performed with the QuantStudio 3 Detection System (ABI, Thermo Fisher). Expression of CDH17 was normalized to 18S rRNA and data analyzed with the Comparative Ct method. Primer sets used: 18S control forward 5′-AAGTCCCTGCCCTTTGTACACA-3′, and reverse 5′-GCCTCACTAAACCATCCAATCG-3’; mouse CDH17 forward 5′-GCTACAGATCTGGATGATCCG-3′, and reverse 5′- ATGTCCTTCACCGAGACCAC-3’; human CDH17 forward 5′- GCCAATCCTCCTGCTGTG-3′, and reverse 5′- GCAACCTGGAGATTGTGAGT-3’; and VP1.5 forward 5′-GGACTTTCCAAAATGTCG-3′ and XL39 reverse 5′-ATTAGGACAAGGCTGGTGGG-3′

### 2.6 Orthotopic PC murine model

PC cells were grown to 90% confluences and suspended in 15% Matrigel in PBS. Suspended cells were then injected into the head of the pancreas in wild-type (WT) C57BL/6 mice at a dose of 2.5 × 10^5^ per mouse.

### 2.7 Proliferation assay

Human PC cells were seeded into 96-well plates at a density of 2 × 10^3^/well. 24 and 48 hours later, cell proliferation was measured with the Proliferation Assay Kit (Promega) according to the manufacturer's instructions.

### 2.8 Colony formation assay

Human PC cells were seeded into 6-well plates at a density of 200 cells/well. 10 days later, cells were rinsed and then stained with 0.05% crystal violet for colony counting.

### 2.9 Wound healing assay

Human PC cells were seeded into 24-well plates at a density of 2 × 10^5^/well. 24 hours later, the insert was removed. The cell-free gap was measured under an optical microscope (Zeiss) at the indicated time points.

### 2.10 Immunohistochemistry (IHC)

4 μm tissue sections were prepared with formalin-fixed and paraffin-embedded tumor tissue. To conduct IHC, tissue sections were de-waxed with xylene and rehydrated with various dilutions of ethanol. Antigens were retrieved with antigen unmasking solution (Vector Laboratories), permeabilized with 0.2% Triton X-100, and blocked with serum. The endogenous peroxidase was quenched with BLOXALL reagent (Vector Laboratories). Lastly, the sections were serially incubated with primary antibodies, secondary antibodies, and DAB peroxidase (HRP) substrate (Vector Laboratories) to develop color. Primary antibodies used are as follows: CDH16 (ab183745; 1:4000), E-cadherin (ab231303; 1:1000), N-cadherin (ab98952; 1:500), and Normal IgG (Mouse - sc-2025; Rabbit - sc-2051).

### 2.11 Statistical analysis

Paired data were analyzed using a 2-tailed paired student's t-test. A *p* value of <0.05 was considered significant.
